# Pollen Organ *Telangiopsis* sp. of Late Devonian Seed Plant and Associated Vegetative Frond

**DOI:** 10.1371/journal.pone.0147984

**Published:** 2016-01-25

**Authors:** De-Ming Wang, Mei-Cen Meng, Yun Guo

**Affiliations:** 1 Key Laboratory of Orogenic Belts and Crustal Evolution, Department of Geology, Peking University, Beijing 100871, China; 2 Science Press, China Science Publishing & Media Ltd., 16 Donghuangchenggen North Street, Beijing 100717, China; 3 Department of Geology, School of Resource Environment and Earth Science, Yunnan University, Kunming 650091, Yunnan Province, China; Institute of Botany, CHINA

## Abstract

Pollen organ *Telangiopsis* sp., associated with but not attached to vegetative fronds, has been collected from the Upper Devonian (Famennian) Wutong Formation, Dongzhi County, Anhui Province, China. Fertile axes with terminal pollen organs are dichotomous for 2–4 times and may be proximally attached by fragmentary pinnules. Pollen organs are synangiate and borne on the top of a short stalk. Synangia are radial in symmetry and each consists of 4–8 elongate microsporangia fused at base. Microsporangia have a longitudinal dehiscence line and show a tapered apex. The associated stem is spiny and bears a vegetative frond which bifurcates once at the basalmost part. Frond rachises possess one order of pinna arranged alternately. Pinnules are borne alternately, planate, highly dissected, and equally dichotomous for 2–3 times. Comparisons among Late Devonian seed plants recognize several branching patterns in the fertile fronds/axes bearing terminal pollen organs. *Telangiopsis* sp. reinforces that the Late Devonian pollen organs are synangiate usually with basally fused microsporangia. It is suggested that the evolutionary divergence of radial and bilateral symmetries of pollen organs may have occurred in the Famennian, when the earliest seed plants evolved planate and sometimes laminate pinnules.

## Introduction

Numerous and highly diversified ovules have been well known from the Famennian of the Late Devonian and they indicate the first major evolutionary radiation of the seed plants or spermatophytes [1–7]. However, the pollen organs of the earliest seed plants in the Famennian are rare and usually incomplete [1, 8], and the vegetative fronds, especially those attached to stem and bearing pinnules, are little known.

Here we report pollen organs (*Telangiopsis* sp.) on fertile axes as well as associated vegetative fronds from the Late Devonian (Famennian) deposits of Anhui Province, China. Based on the well preserved specimens and comparisons with other relative taxa, we summarize the characters of Famennian pollen organs, stems, vegetative fronds and pinnules. Regarding the earliest seed plants, we also briefly discuss the branching pattern of pollen organ–bearing fronds or axes, evolution of synangiate symmetry, planation of vegetative pinnae, and morphology of pinnules.

## Material and Methods

Fossil plants were collected from the Upper Devonian Wutong (Wutung) Formation (Leigutai Member) at the Xiangkou section (GPS data 30°03′57′′N and 116°47′26′′E), Xiangyu Town, Dongzhi County, Anhui Province, China. Details of the locality and stratigraphy were provided in previous studies [8, 9]. Wutong Formation, widespread in the lower reaches of the Yangtze River including Anhui, consists of Guanshan Member (quartzose sandstone and conglomerate) and overlying Leigutai Member (quartzose sandstone interbedded with mudstone) [10]. Assemblages of plants, spores, fish and conchostracans indicate that the Wutong Formation is Famennian in age [10–12]. From the eighth bed of the Wutong Formation (Supplementary Fig 1 in ref. 8) and in the mudstone of the Leigutai Member, we obtained about 80 pollen organs associated with 15 vegetative branches, which were preserved as impressions and compressions. These vegetative branches represent only one type of frond and they are found associated with pollen organs. At the Xiangkou section, some pollen organs occur closely with ovules in the same horizon. The progymnosperm *Archaeopteris halliana* [9] and seed plant pollen organ *Placotheca minuta* [8] have been previously studied in Wutong Formation of the same section. During the fieldwork, no permits were required for the described study, which complied with all relevant regulations.

Specimens examined in this study include PKUB14801a, b, PKUB14807, PKUB14813, PKUB14814, PKUB14816b, PKUB14817, PKUB14823, PKUB14840a, PKUB14841b, PKUB14842a, b, PKUB14880, PKUB14882 and PKUB14887. All specimens have been deposited in Department of Geology, Peking University, No.5 Yiheyuan Road, Haidian District, Beijing, China. Steel needles were used to expose some fertile axes, pollen organs, vegetative pinnae and pinnules. All photographs were made with an Olympus digital camera and an Olympus microscope, and they were prepared with software Photoshop CS3 and CorelDRAW X4.

## Results

### Fertile axes and pollen organs

**Fig 1:**
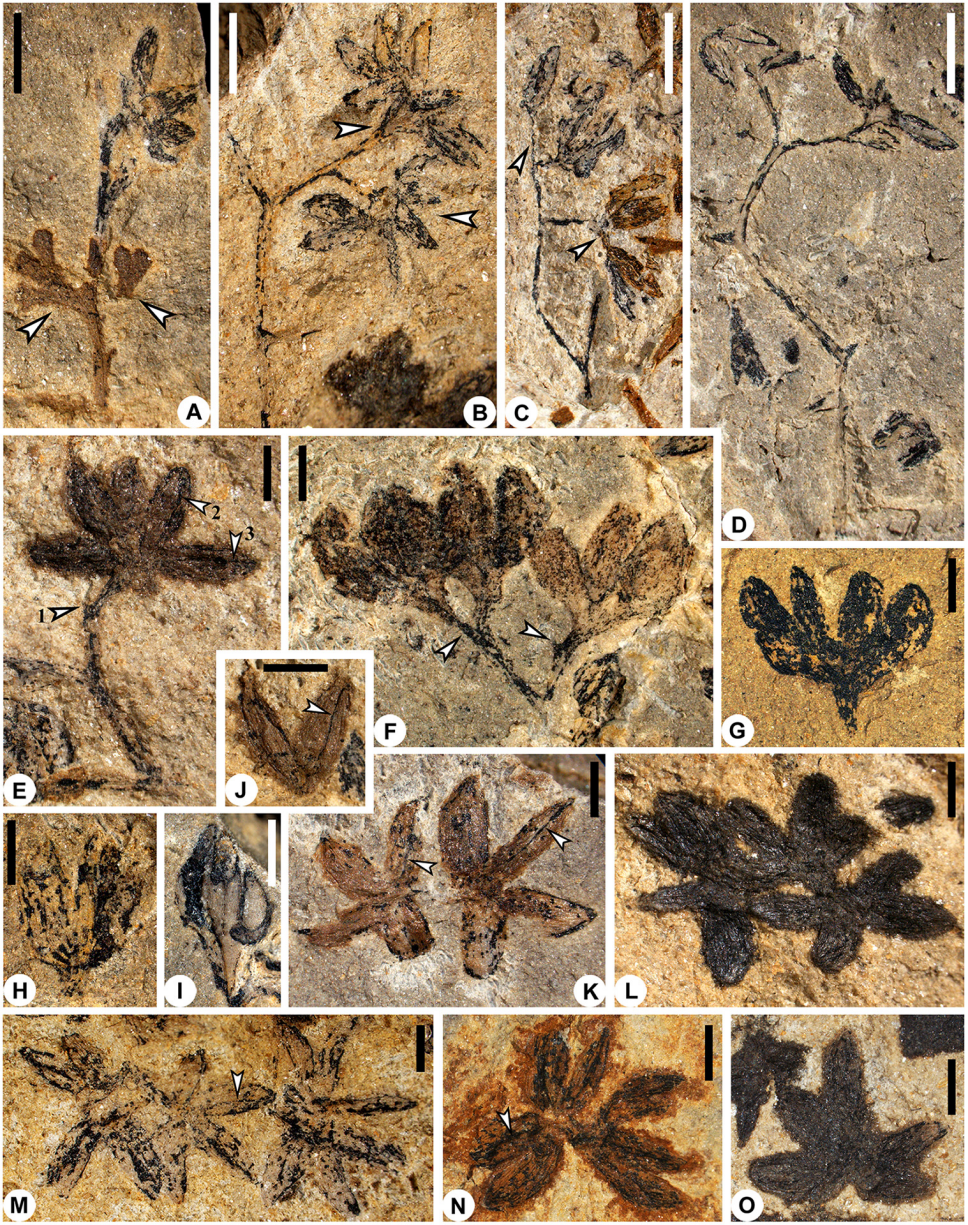
Fertile axes and synangiate pollen organs of *Telangiopsis* sp. A, Twice dichotomous axes attached by one pollen organ and two planate pinnules (arrows) (PKUB14817). B, Four times branching axes with three terminal pollen organs. Upper and lower arrows indicating two pollen organs and a single one, respectively (PKUB14801a). C, Thrice dichotomous axes with two pairs of terminal pollen organs (arrows) (PKUB14842a). D, Four times branching axes terminated by fragmentary pollen organs (PKUB14813). E, Dichotomous axes with one terminal pollen organ preserved. Arrow 1 indicating probably broken point of another pollen organ, arrows 2 and 3 dehiscence line on microsporangium (PKUB14801a). F, Paired pollen organs (arrows) terminating twice dichotomous axes. Arrow 2 indicating broken point of a probable pollen organ (PKUB14816b). G–I, Lateral view of synangium with basally fused microsporangia (PKUB14887, PKUB14817 and PKUB14814, respectively). J, Two microsporangia showing dehiscence line (arrow) (PKUB14807). K–O, Synangia with basally fused microsporangia showing ventral surface. K, Two pollen organs. Arrows showing dehiscence line on microsporangium (PKUB14841b). L, M, Three pollen organs and dehiscence line (arrow) (PKUB14801b and PKUB14801a, respectively). N, O, One pollen organ and dehiscence line (arrow) (PKUB14840a and PKUB14801b, respectively). A–D, scale bars = 2 mm. E–O, scale bars = 1 mm.

**Fig 2:**
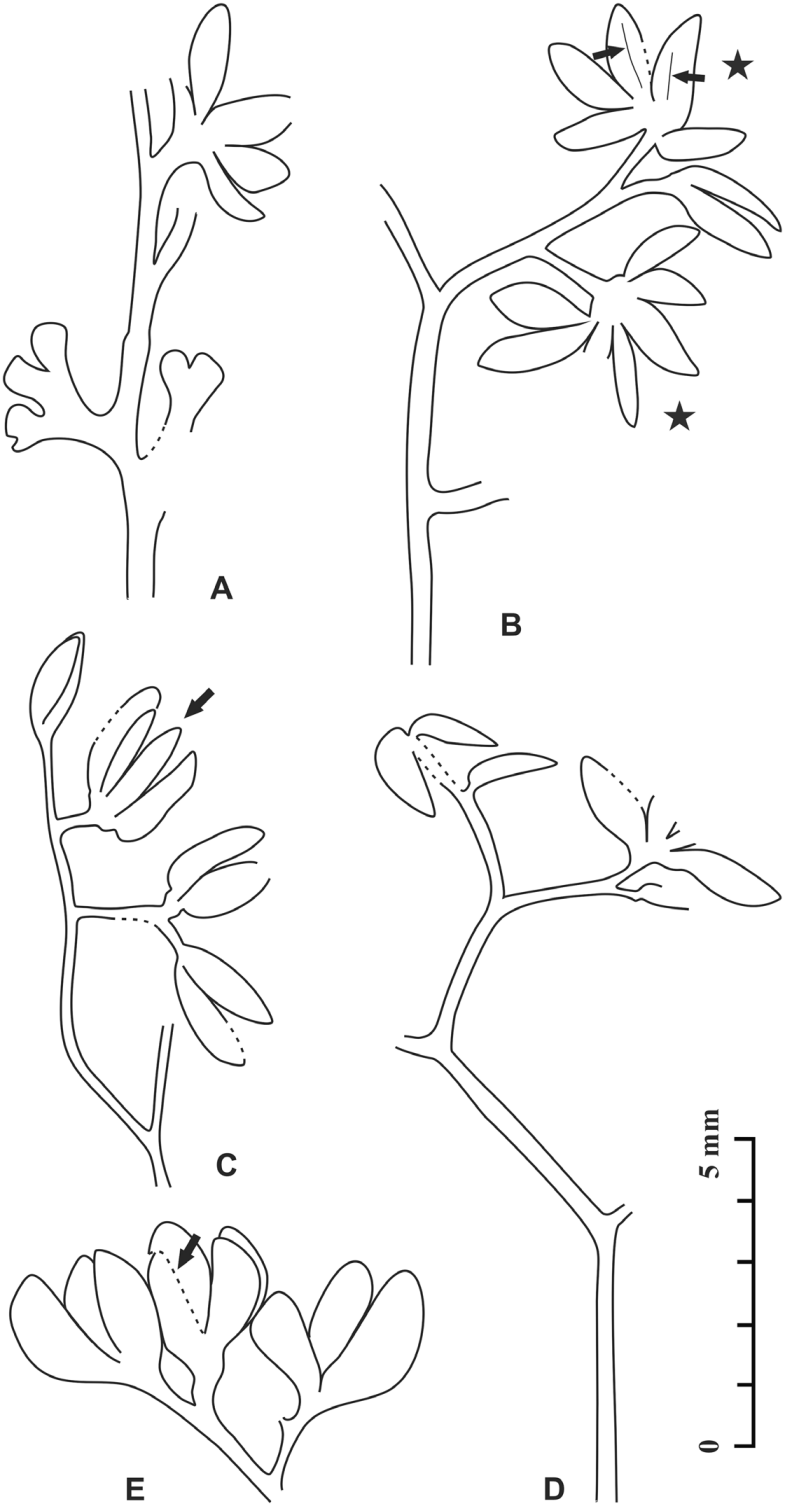
Fertile axes and terminal pollen organs of *Telangiopsis* sp. A–E, Line drawings of Fig 1A–1D and 1F, respectively. B, Arrows indicating dehiscence line of two microsporangia and stars two pollen organs. C, Arrow indicating a pollen organ. E, Arrow showing limit of two overlapped microsporangia.

Fertile axes are smooth, up to 13.7 mm long and 0.2–0.5 mm wide, and dichotomous at 30–95° for 2–4 times (Figs 1A–1F and 2). Their internodes (1.0–4.2 mm long) and width reduce distally. The lower part of a branch is attached by a relatively complete pinnule (Fig 1A, left arrow, Fig 2A) and a fragmentary one (Fig 1A, right arrow), which are ca. 2.0 mm long and 2.2 mm wide and borne alternately. The complete pinnule is planate and appears to divide twice into four units, with each unit being 0.3–0.8 mm long and ca. 0.5 mm wide.

Some pollen organs terminate fertile axes (Figs 1A–1F and 2), whereas the others are detached (Fig 1G–1O). Individual pollen organs are borne distally on a short stalk (Figs 1A–1H and 2) and, in some cases, they appear to occur in pairs (Fig 1B, upper arrow, Fig 1C, lower arrow, Fig 1F, left arrow, Fig 2B, 2C and 2E). Probably due to the preservation or lacking counterpart of specimen, one pollen organ probably in a pair and its stalk are sometimes missing (Fig 1E, arrow 1, Fig 1F, right arrow and Fig 2E). In other cases, however, the pollen organs seem to be borne singly on the top of a short part of fertile axis (Fig 1B, lower arrow, Fig 1C, upper arrow, Fig 2B, lower star and Fig 2C). The pollen organs in surface view are radially symmetrical and lack a pad or cushion (Fig 1K–1O).

In lateral view (Figs 1A–1I and 2) and surface view (Fig 1K–1O), the microsporangia of a single pollen organ, albeit free in lateral and distal parts, are basally fused. Therefore, the pollen organs are synangiate in structure. Each completely and well preserved synangium consists of 4 (Figs 1C, 1F–1I, 1K and 2C, arrow and Fig 2E, arrow) or 5 (Fig 1A and 1B, upper arrow, Figs 1E, 1L, 1M, 1O, 2A and 2B, upper star) or 6–8 (Fig 1B, lower arrow, Figs 1N and 2B, lower star) microsporangia (Figs 1A–1C, 1E–1I, 1K–1O, 2A–2C and 2E). Because of preservation, the synangia probably in a pair may have different numbers of microsporangia (Figs 1B, 1C, 2B and 2C).

**Fig 3:**
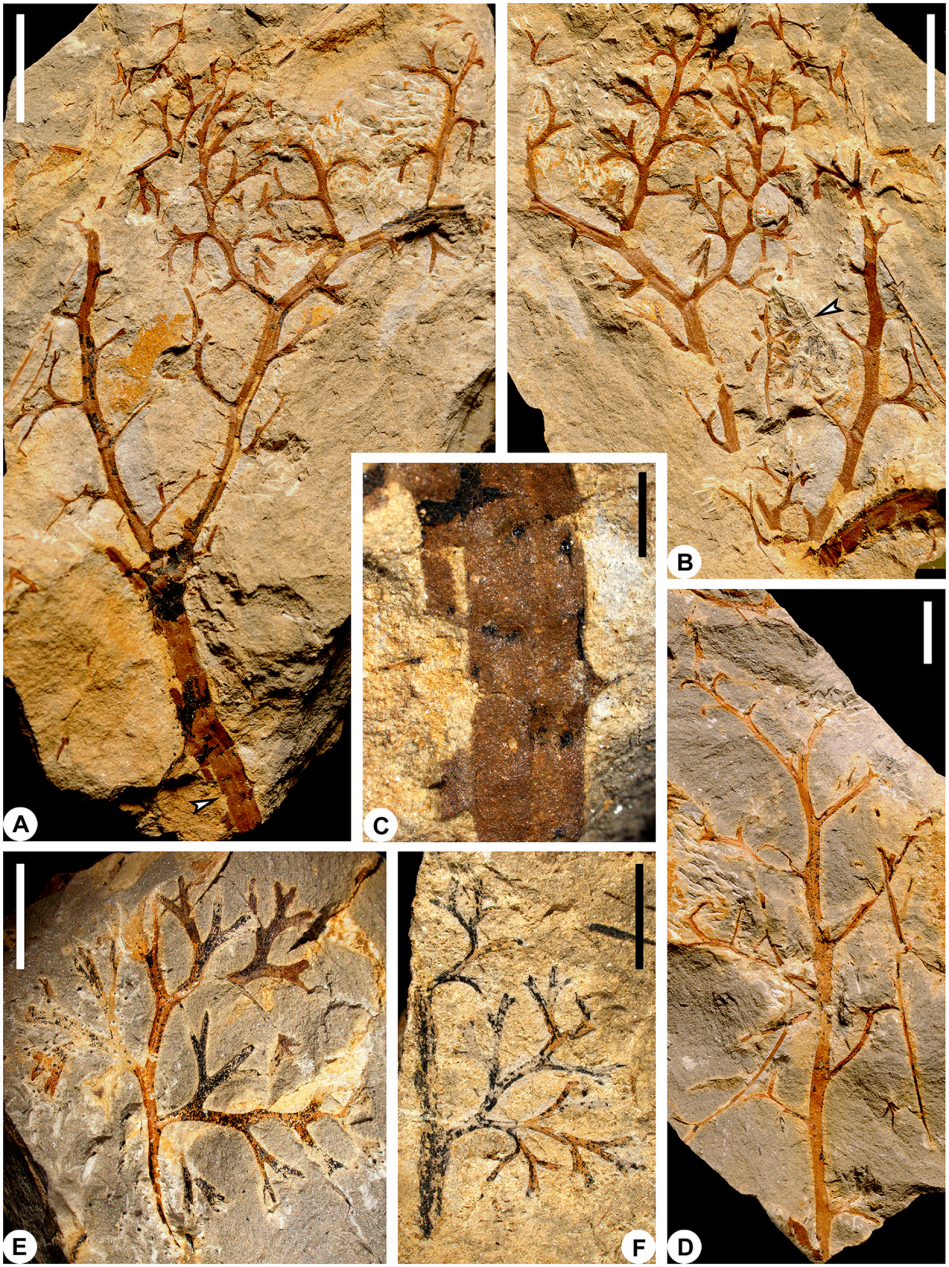
Stem and/or vegetative fronds of *Telangiopsis* sp. A, B, Part and counterpart of a specimen showing stem attached by proximally bifurcate frond. Frond rachises bearing pinnae and highly dissected pinnules in alternate arrangement (PKUB14842b, PKUB14842a). A, Arrow indicting part of stem enlarged in C. C, Enlargement of arrowed part of A, showing spines and their scars on stem. D, Frond rachis with alternately arranged pinnae (PKUB14882). E, F, A piece of frond rachis bearing pinnae and planate pinnules (PKUB14880, PKUB14823). A, B, scale bars = 1 cm. C, scale bar = 2 mm. D–F, scale bars = 5 mm.

The microsporangia are elongate and have a tapered tip. The appearance of a round tip is due to the oblique orientation of microsporangium in the rock matrix (e.g., Fig 1K, lower part, Fig 1L, lower part and Fig 1O). Parallel striations are evident on the surface of several microsporangia (Fig 1J, 1L and 1N). The longitudinal dehiscence line is sometimes visible on the sporangial wall toward the pollen organ center (Fig 1E, arrows 2 and 3J, arrow, Fig 3K, arrows, Fig 3M, arrow, Fig 3N, arrow and 2B, arrows), and these microsporangia show the ventral surface. In a single synangium, the microsporangia directing toward the pollen center may also demonstrate the ventral surface, although their dehiscence line is invisible (Fig 1L and 1O). Sometimes, it is difficult to identify the dorsiventrality of the microsporangia (Fig 1F–1I). Measurements of the synangia, stalks and microsporangia are included in Table 1.

**Table 1:** Characters of the Late Devonian pollen organs.

Taxa	Synangium				Stalk		Microsporangium				
	length (mm)	width (mm)	symmetry	pad	length (mm)	width (mm)	number per synangium	fusion	apex	length (mm)	width (mm)
*Cosmosperma polyloba* [6]	2.2–2.4	2.4–2.9	?	absent	1.0	0.2–0.3	6–8	basal	tapered	2.3	0.6–0.7
*Elkinsia polymorpha* [13]	2.0–3.0	1.5–2.0	radial	absent	?	?	6–8	basal	round acute	1.6–2.4	0.2–0.8
*Kongshania synangioides* [15]	2.0–3.0	1.5–2.0	?	present	0.6–1.0	0.2–0.3	6	basal	pointed	4.7–8.0	1.1–1.8
*Placotheca minuta* [8]	1.6–2.2	0.8–1.9	bilateral	present	?	?	up to 60	basal lateral	tapered	0.9–1.3	0.1
*Telangiopsis* sp. [18]	>3	?	radial	absent	?	?	4, >5	basal	hooked	1.6–1.8	0.6–0.7
*Telangium schweitzeri* [14]	>3.9	up to 1.6	bilateral	absent	0.7–1.1	0.1–0.3	8	basal	beaked	3.2	0.1–0.4
*Telangiopsis* sp.	2.1–2.9	1.4–2.6	radial	absent	0.5–1.3	0.2–0.4	4–8	basal	tapered	1.2–2.7	0.4–0.9

**Notes:**?, unknown.

### Stem, vegetative fronds and pinnules

**Fig 4:**
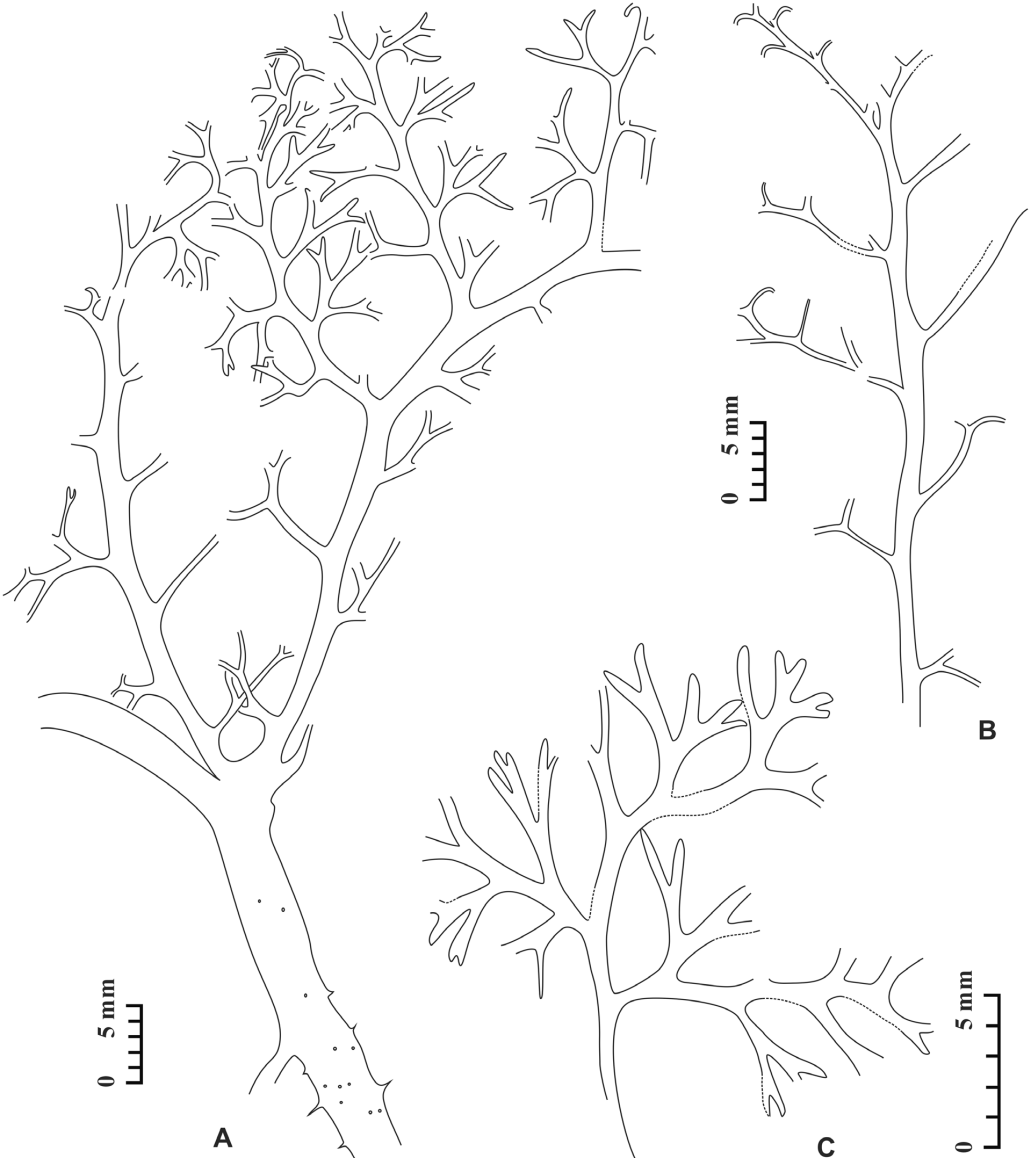
Stem and/or vegetative fronds of *Telangiopsis* sp. A, Line drawing of Fig 3A and 3B in combination. B, C, Line drawings of Fig 3D and 3E, respectively.

In the same bedding plane, a piece of stem with a vegetative frond (Fig 3A) is closely associated with fertile branches bearing terminal pollen organs (Fig 3B, arrow). The fertile portion is enlarged in Fig 2C and the image has been rotated. The stem curves in the upper part (Fig 4A) and its mid–lower part bears some spines (Fig 3A, arrow), which are 0.6–1.2 mm long and 0.3–0.9 mm wide at base (Figs 3C and 4A). These two parts of stem are different in width. The vegetative frond bifurcates basally once at 60° to produce two slightly curved rachises of the same width (Figs 3A and 4A), which are narrower than the stem. There is only one order of pinna rachis and these rachises occur alternately and at 40–90° on the frond rachis (Figs 3A, 3B, 3D–3F and 4). The number of pinnae on a single frond rachis is up to 14 (Figs 3D and 4B). The interval between two adjacent pinna rachises is 2.4–7.0 mm and may decrease acropetally. The pinnae are 11–22 mm long and 8.6–12 mm wide. No protrusions such as spines are visible on the frond or pinna rachises.

The pinna rachis possesses up to eight pinnules in alternate arrangement (Figs 3A, 3B, 3E, 3F, 4A and 4C). The distance between two adjacent pinnules ranges from 1.5 mm to 3.1 mm. Nonlaminate pinnules are borne at 50–90°, planate and highly dissected. Each pinnule equally dichotomizes at 20–75° for two or three times to produce four or eight units. These units are 0.5–3.0 mm long and 0.2–0.5 mm wide and distally tapered. Table 2 shows the measurements of stem, frond rachises, pinna rachises and pinnules.

**Table 2:** Characters of stems and vegetative fronds of the Late Devonian seed plants.

Taxa	Stem			Frond rachis			Pinna rachis		Pinnule			
	length (mm)	width (mm)	surface	length (mm)	width (mm)	branch times	length (mm)	width (mm)	length (mm)	width (mm)	planate laminate	shape
*Elkinsia polymorpha* [13]	up to 530	3.0–9.0	smooth	up to 350	1.0–10	3–4	40–170 (ppr, spr)	0.5–10(ppr, spr)	2.0–15	2.0–10	planate laminate	club
*Kongshania synangioides* [15]	61–85	4.0–18	smooth	up to 130	1.1–2.8	?	up to 104 (ppr) 10–40 (spr) 6.0–10 (tpr)	0.8–1.0 (ppr) 0.5–0.8 (spr) 0.4–0.5 (tpr)	1.2–3.3	1.5–2.5	laminate	wedge tongue
*Laceyahibernica* [20]	270	20–47	smooth	over 155	1.5–21	1	up to 33	0.7–2.1	?	?	?	?
*Yiduxylon trilobum* [21]	up to 120	7.2–20	smooth	up to 60	4.8–10	1	up to 76 (ppr) up to 60 (spr)	1.8–2.6 (ppr) 0.8–1.3 (spr)	up to 22	19	planate	dissected
*Telangiopsis* sp.	>40	2.8–4.0	spiny	up to 47	0.6–1.7	1	9.0–21	0.4–0.8	3.6–7.0	4.2–6.0	planate	dissected

**Notes:**?, unknown; ppr, primary pinna rachis; spr, secondary pinna rachis; tpr, tertiary pinna rachis.

## Comparisons with early seed plants

### Fertile axes with terminal pollen organs

Fertile axes of *Telangiopsis* sp. are dichotomously branched and the lower part of a fertile branch bears two planate pinnules. Although the pinnules (Figs 1A and 2A) are smaller than those on the vegetative pinna rachises, they present the same shape and mode of division and arrangement, or perhaps have been distally truncated. Where known, however, the fertile axes of *Elkinsia* [13] and *Telangium schweitzeri* [14] are cruciately branched, and those of *Kongshania* as reconstructed in Text-fig 3 of [15] are pinnately arranged. Furthermore, the entire fertile fronds of *Elkinsia* lack pinnules.

### Pollen organs

The fossil genus *Telangiopsis* refers to generally simple pollen organs preserved as compressions, which are morphologically similar to the anatomically preserved genus *Telangium* [16]. *Telangiopsis* is characterized by radially symmetrical synangia terminating dichotomous or monopodial axes, and stalked microsporangia fused only at base [16, 17]. *Telangiopsis* sp. in this paper conforms to such diagnostic features.

Prior to this study, six Late Devonian seed plants have been known for pollen organs (Table 1). *Telangiopsis* sp. from Xiangkou section resembles them in the size of synangia, stalks and microsporangia (except for microsporangium size of *Kongshania* and *Placotheca*), number of microsporangia (4–8) per synangium (except for *Placotheca*), and basal fusion of elongate microsporangia. Nevertheless, *Kongshania* from China has larger microsporangia [15]; *Placotheca* from China is characterized by bilaterally symmetrical synangia, which possess a pad and much more and smaller microsporangia fused basally and somewhat laterally [8]; the microsporangia of *Telangiopsis* sp. from England bear a hooked tip [18]; in *Telangium schweitzeri* from Ireland, the synangia are bilaterally symmetrical and the microsporangia bear a beaked tip [14]. Pollen organs of *Cosmosperma* from China and *Telangiopsis* sp. are very close in dimensions and structures. However, *Cosmosperma* [6] lacks information on fertile axes and vegetative fronds. This plant has larger and more complex pinnules, which are 11.0–13.3 mm long and 10.0–13.0 mm wide and include alternate units. *Elkinsia* from USA and *Telangiopsis* sp. share similar pollen organs. In contrast, the synangia of *Elkinsia* are borne terminally on cruciate branches and the vegetative fronds may possess laminate pinnules [13]. More importantly, at the Xiangkou section, *Telangiopsis* sp. is closely preserved with a kind of ovule. This ovule is now under study and clearly represents a new genus. If the pollen organs and ovules from this section belong to the same taxon, *Telangiopsis* sp. could be more easily differentiated from the pollen organs of the other Late Devonian seed plants.

In the Carboniferous, the comparatively better known species of *Telangiopsis* include Mississippian *T*. *arkansanum* from USA, *T*. *bifidum* and *T*. *affine* from UK and Ireland, *T*. *nonnae* from Russia and Pennsylvanian *T*. *nutans* from France and Belgium [16, 17, 19]. Pollen organs of *T*. *arkansanum* may terminate monopodial axes and the individual synangia are only ca. 1.0 mm long and 0.8 mm wide, whereas the synangia of *Telangiopsis* sp. in this paper terminate dichotomous axes and are larger. Terminal synangia of *T*. *nonnae* and *T*. *nutans* are borne on monopodial axes. Differing from those of *Telangiopsis* sp., the individual synangia of *T*. *bifidum* consist of more (up to 25) microsporangia and the synangia of *T*. *affine* are larger (2.5–3.5 mm long and 2.8–3.0 mm wide).

### Stems with vegetative fronds

In Late Devonian seed plants, there have been no taxa showing the attachment of vegetative fronds to pollen organs. Where known, the vegetative fronds are associated with the pollen organs [13, 15], as they are in our material. Although not found attached, one type of vegetative frond is closely and consistently associated with the pollen organ. In this low-diversity flora, the frond suggests a former connection to the pollen organ. Furthermore, the pinnules on the fertile axes terminated by pollen organ are similar to those on the vegetative fronds in the shape and pattern of division and arrangement. If pollen organs of *Telangiopsis* sp. and associated fronds belong to the same plant, the following relative comparisons and discussion can be made.

The stems and vegetative fronds have been previously reported in four Late Devonian seed plants (Table 2). These genera and *Telangiopsis* sp. from Xiangkou section have alternate arrangement of pinnae and pinnules. *Telangiopsis* sp. mainly differs from them in spines and width of stems, basalmost bifurcation (at the attaching point of frond to stem) and width of frond rachises, and width of pinna rachises. The frond rachis bifurcation of *Elkinsia*, *Laceya* and *Yiduxylon* occurs more or less above the frond attachment [13, 20, 21]. In addition, *Elkinsia* has 3–4 divisions of frond rachis, two orders of pinnae and laminate pinnules of club shape; *Kongshania* possesses three orders of pinnae and laminate pinnules of wedge/tongue outline; *Yiduxylon* bears two orders of pinnae and larger pinnules.

## Discussion

### Types of branches with terminal pollen organs/fructifications

Carboniferous (Mississippian) seed plants include three types of fertile fronds terminated by pollen organs or fructifications [17, 22]: 1) pinnate branches possessing both synangia and pinnules (*Rhodea* type); 2) trifurcate frond rachis producing a median dichotomous fertile rachis (*Diplopteridium* type); 3) frond rachis with basal part bearing two–dimensional vegetative pinnae/laminate pinnules and distal fertile part that are highly divided but have independent sporangia (*Rhacopteris*/*Triphyllopteris* type). Among Late Devonian (Famennian) seed plants currently known for axes or fronds with terminal pollen organs, the cruciate branching evidenced by *Elkinsia* and *Telangium schweitzeri* is absent in the Mississippian taxa. Fertile axes of *Kongshania* and *Rhodea* type fertile frond share a pinnate arrangement. At present, there is no record of *Diplopteridium* type fertile frond in Famennian seed plants.

Except for the planation of pinnules and less dichotomies in the distal part, the fertile axes of *Telangiopsis* sp. in this paper somewhat resemble *Rhacopteris*/*Triphyllopteris* type fertile frond in the position of pinnules and pollen organs. There is controversy over the affinities of *Rhacopteris* and *Triphyllopteris* [3]. However, the foliage anatomy of *Rhacopteris* and the fertile fructifications of *Triphyllopteris* suggest seed plant characters [23, 24]. If so and considering the differences with *Telangiopsis* sp., *Rhacopteris*/*Triphyllopteris* type fertile frond is derived in the lamination of pinnules and complexity of distal fructifications.

### Pollen organs

It has been suggested that the earliest seed plants in the Famennian possess synangiate pollen organs, which generally have a few basally fused microsporangia and lack a synangial pad; these synangia clearly differ from the fructifications of Middle to Late Devonian (Givetian to Frasnian) ancestral aneurophyte progymnosperms, which consist of many independent and pinnate sporangia [6]. Such suggestions are supported by the characters of pollen organ of *Telangiopsis* sp.

Synangiate pollen organs of the Carboniferous seed plants are characterized by radial or bilateral symmetry [17, 25–27]. Based on comparative morphology of aneurophytes and available evidence in the Carboniferous, the radial symmetry of synangia has been considered primitive [14, 25]. Famennian pollen organs are radially or bilaterally symmetrical (Table 1). Thus, they probably represent a potential stage preceding the evolutionary divergence of synangial symmetries manifested in younger spermatophytes.

Longitudinal dehiscence along the inner facing wall of a microsporangium has been found in the Late Devonian pollen organs of *Telangium schweitzeri* [14], and it is now observed in *Telangiopsis* sp. As stated by many researchers [25, 27–29], the microsporangium dehiscence line of early seed plants indicates that the pollen was shed toward the pollen organ center.

### Vegetative fronds and pinnules

Compared to the ancestral aneurophytes, the early seed plants demonstrate derived morphological features such as bipartite fronds [22]. *Telangiopsis* sp. conforms to this feature in that the frond rachis is proximally bifurcate. Its vegetative branching system may indicate the upper part of a plant because of slender stem, frond and pinnae rachises as well as only one order of pinna.

The pinnae and/or pinnules of the Late Devonian seed plants are arranged in one plane [6]. This character is now confirmed by *Telangiopsis* sp. Planate and laminate pinnules are widespread in the Carboniferous seed plants [3, 26, 30–32]. The presence of such pinnules can be traced back to Famennian, when the primitive taxa exhibit pinnules that are often planate (highly dissected) or sometimes laminate with lobes. These pinnules of different shapes may occur in the same plant (*Elkinsia*) [13].
